# Clinical characterization of *Helicobacter pylori* infected patients 15 years after unsuccessful eradication

**DOI:** 10.1371/journal.pone.0238944

**Published:** 2020-09-23

**Authors:** Oddmund Nestegard, Kay-Martin Johnsen, Sveinung W. Sørbye, Fred-Arne Halvorsen, Tor Tønnessen, Eyvind J. Paulssen, Kjetil K. Melby, Rasmus Goll, Jon Florholmen

**Affiliations:** 1 Department Gastroenterology, Vestre Viken Hospital, Hønefoss, Norway; 2 Department of Clinical Medicine, Research Group of Gastroenterology and Nutrition, UiT The Arctic University of Norway, Tromsø, Norway; 3 Department of Pathology, University Hospital of North Norway, Tromsø, Norway; 4 Department Gastroenterology, Vestre Viken Hospital, Drammen, Norway; 5 Department of Microbiology, University Hospital Oslo and University of Oslo, Oslo, Norway; Nitte University, INDIA

## Abstract

**Background and aims:**

Patients that have failed therapy for *Helicobacter pylori* (*H*. *pylori*) infection are incompletely characterized. The aim of this study was to characterize a *H*. *pylori* treatment resistant cohort compared to the cohorts of newly diagnosed, earlier eradicated and non-infected.

**Material and methods:**

Patients were selected from routine referrals to the Endoscopy units at three different Norwegian hospitals. In all four cohorts, gastric biopsies were scored according to the Sydney classification, and symptoms according to the Gastrointestinal Symptom Rating Scale score, including sub-scores for upper gastrointestinal symptoms and functional bowel symptoms. Patients in the *H*. *pylori* resistant group were treated with a triple therapy regimen that consisted of levofloxacin, amoxicillin and a proton pump inhibitor.

**Results:**

We included 185 patients, 42 *H*. *pylori* treatment resistant, 50 newly diagnosed, 61 previously *H*. *pylori* eradicated and 32 never infected. The treatment-resistant cohort had higher scores for upper gastrointestinal symptoms and functional bowel symptoms compared to the other groups except for the group being never *H*. *pylori* infected. The *H*. *pylori* resistant patients had lower Sydney scores than patients with newly diagnosed *H*. *pylori* infection. The triple combination showed a high efficacy of 91% to eradicate *H*. *pylori*.

**Conclusions:**

Patients with treatment-resistant *H*. *pylori* infection had more gastrointestinal symptoms, but a lower Sydney score than patients with newly diagnosed infection. A treatment regimen including levofloxacin showed a high efficacy in eradicating *H*. *pylori* in patients that previously had failed eradication treatment.

## Introduction

*Helicobacter pylori* (*H*. *pylori*) has been identified as the main pathogenic factor for gastric and duodenal peptic ulcer.[1,2] Treatment of *H*. *pylori* infection cures most of the patients with ulcer, and the disease is no longer a chronically recurrent and disabling condition in Western world. However, in approximately 50% of the population worldwide, the infection is still a great problem, especially in underdeveloped countries.[3,4]

Initially, the most effective eradication regimes gave an efficacy >90%.[5] Unfortunately, during the 30 years of treatment one has observed a growing antimicrobial resistance of *H*. *pylori*[6], including resistance to clarithromycin and metronidazole, proposed to be a consequence of increased antibiotic use in the general population. Clarithromycin resistance has especially been of major negative impact as it was a main constituent of the recommended first line triple therapy. Metronidazole resistance is highly prevalent but is possible to overcome to some extent.[7] Thus, an increasing prevalence of resistance has been observed.[8,9] In areas with high occurrence of both clarithromycin and metronidazole resistance, a bismuth-containing quadruple therapy is recommended.[10] Despite this, a cohort of patients with treatment-resistant *H*. *pylori* has accumulated even in areas with low prevalence of antibiotic resistance, like Norway.[9] These patients will have a persistent gastric inflammation and a risk of recurrent ulcer disease and gastric cancer. There are few reports of the clinical or microbiological characteristics of this cohort, but some data has been presented in the search for a new effective antibiotic therapy, including levofloxacin.[11–13]

The goal of this study was to characterize a cohort with treatment-resistant *H*. *pylori* infection, and make clinical comparisons to patients with newly diagnosed *H*. *pylori* infection, previously successfully eradicated *H*. *pylori*, and a non-infected cohort. Finally, we wanted to evaluate the response to treatment with levofloxacin.

## Materials and methods

### Study population, enrolment and patient flow

Patients were recruited in the period 1990–2012 for screening in the **C**h**r**onical **I**nfection of **He**licobacter **P**ylori (CRIHEP) study from three different gastrointestinal units at Norwegian hospitals: University Hospital of North Norway, Tromsø; Vestre Viken Hospital, Ringerike; and Vestre Viken Hospital, Drammen; Norway.

Four groups were invited to participate: The treatment-resistant *H*. *pylori* group (n = 42) were patients who had two or more unsuccessful treatment attempts for *H*. *pylori* infection. They were selected from medical records from 1990 to 2012 at the three different hospitals, and were invited to participate by a mail request. Responders were included after a new gastroscopy examination. The three additional patient groups were outpatients referred to the Endoscopy unit for gastroscopy: The newly diagnosed *H*. *pylori* infection group (n = 50) were included after positive findings of *H*. *pylori* infection at the endoscopic examination, the group of previously successfully treated *H*. *pylori* infection 5–20 years ago (n = 61), and the group of patients without any diagnosed *H*. *pylori* infection ever (n = 32) also underwent upper endoscopy. Patients with severe disease and those who were non-compliant were excluded.

Biopsies were obtained at endoscopy from the gastric corpus and pyloric antrum for histological examination and *H*. *pylori* rapid urease test.

Patients in the treatment-resistant *H*. *pylori* group were treated with oral omeprazole 20 mg b.i.d, amoxicillin 1 g b.i.d. and levofloxacin 500 mg b.i.d. for 10 days. Those with allergy to penicillin received metronidazole 500 mg t.i.d instead of amoxicillin.

Patients with previously untreated *H*. *pylori* infection and findings of peptic ulcer, erosive gastritis and/or erosive duodenitis were treated with oral omeprazole 20 mg b.i.d., amoxicillin 1 g b.i.d. and clarithromycin 500 mg b.i.d. for seven days. In patients with allergy to penicillin, metronidazole was used instead of amoxicillin as described above.

All subjects that were given treatment for *H*. *pylori* infection underwent a follow-up gastroscopy after 3–6 months upon which biopsies were obtained for the various examinations described above.

All patients gave an oral and written consent before enrolment in the study. The study was approved by the Regional Committee for Medical and Health Research Ethics (REC-North, ID:2009/2176-11), including approved the storage of biological material.

### Symptom scores

All patients filled out the validated Gastrointestinal Symptoms Rating Scale (GSRS) questionnaire.[14] The GSRS includes 15 questions about symptoms where the answers are rated on a 7-graded scale, 1 representing absence of symptoms and 7 most severe symptoms.

To compare the gastrointestinal complaints among the four groups, comparisons were made between the various dimensions of the GSRS score, and yes/no according to reflux disease, dyspepsia and functional bowel symptoms according to their definitions.

For upper gastrointestinal symptoms, the questions were divided into 5 clinically relevant dimensions of the GSRS: abdominal pain, indigestion (increased flatus), reflux, diarrhoea and constipation, and the total score based on a study of patients referred to upper endoscopy for dyspepsia according to Dimenas et al. and Reviecki et al.[15,16] (Table 2)

The GSRS questionnaire defines ‘‘reflux syndrome” by asking about ‘‘heartburn” or ‘‘acid reflux”. The resulting ‘‘reflux syndrome” score is defined as the mean score of 2 or more of the two items heart burn and acid reflux in GSRS registration according to the references, and also to the Montreal definition from 2006 (Table 3).[14–17] The questions defining dyspepsia were the GSRS dimensions: “Have you had abdominal pain located in the upper abdomen for at least one week”, and “Have you ever had heartburn or acid regurgitation almost daily for at least one week”. A positive answer to at least one of the questions defined dyspepsia as used by Asfeldt et al.[18] (Table 3)

The dimensions most relevant for functional bowel symptoms (FBS) were the ten GSRS questions: abdominal pain, hunger discomfort, borborygmus, abdominal distension, flatulence, constipation, diarrhea, loose stool, urgency and incomplete evacuation. These are the GSRS dimensions that closest resemble irritable bowel disease and has been developed and validated by Wicklund et al.[19] A cut-off value of ≥22 defined a positive diagnosis of functional bowel symptoms based on best comparison (κ = 0.535) described in a previous study from our research group.[20] (Table 3).

### Registrations and analyses

Clinical data including ongoing medication were registered. Biopsies were examined with the rapid urease test for on-site *H*. *pylori* diagnosis. Histological examination (hematoxylin-eosin staining) including detection was performed by one person (SWS) for *H*. *pylori* diagnosis and Sydney classification. A positive *H*. *pylori* status was based on a positive result for at least one of the two tests (*H*. *pylori* rapid -urease test and histological test). Moreover, a negative *H*. *pylori* status was indicated when both tests were negative.

### Statistics

Statistical analyses were performed to evaluate difference between *H*. *pylori* groups in upper gastrointestinal symptoms defined by mean score of the five dimensions of GSRS questionnaire. For analyzing differences between two groups, pairwise Mann-Whitney tests were performed. As a global test for testing differences between groups a non-parametric method, Kruskal-Wallis one-way ANOVA was performed.

To detect potential confounders for reflux, dyspepsia and functional bowel syndrome symptoms, variables were tested in a logistic regression model using an Omnibus logistic regression model. The following variables were tested: *H*. *pylori*, sex, age, body mass index, snuff and smoking habits, and use of platelet inhibitors, NSAIDs, PPI, H2 blockers as well as *H*. *pylori*. Variables with p-values ≤0.10 were included in the multivariate models. Multivariate analyses were stratified for sex and the use of PPI and H2 blockers.

All statistical analyses was carried out in IBM SPSS Statistics 24 (IBM Corporation, Armonk, New York, USA).

## Results

### Patients included

A total of 185 patients, 165 in Tromsø, 18 in Drammen and 2 in Hønefoss, were included in the study. The baseline characteristics of the four study groups are shown in Table 1. Thirty-six of the 42 patients in the *H*. *pylori* resistant group, and 40 of the 50 patients in the newly *H*. *pylori* diagnosed group were treated according to the protocol. The remaining patients refused to participate in the treatment protocol.

**Table 1:** Baseline characteristics of the four different study groups. Values are presented as number (ratio (%)) or mean (95% CI). For further details, see text.

*H*. *pylori* patient groups	Resistant n = 42	Newly diagnosed n = 50	Eradicated n = 61	Never infected n = 32
Age (years)	59 (56–63)	51 (46–56)	62 (59–65)	52 (47–57)
BMI (kg/m2)	26 (25–28)	27 (25–28)	26 (25–28)	25 (24–27)
Sex				
Female	26 (62%)	28 (56%)	31 (51%)	21 (66%)
Male	16 (38%)	22 (44%)	30 (49%)	11 (34%)
Tobacco use				
Non-smoker	30 (71%)	40 (80%)	35 (57%)	21 (68%)
Current smoker	12 (29%)	10 (20%)	26 (43%)	10 (32)
Non-snuffer	42 (100%)	47 (94%)	60 (98%)	30 (97%)
Snuffer	0 (0%)	3 (6%)	1 (2%)	1 (3%)
Medication use				
H2RA	8 (19%)	4 (8%)	8 (13%)	2 (7%)
PPI	17 (40%)	7 (14%)*	23 (38%)	13 (42%)
PI	6 (14%)	7 (14%)	13 (21%)	3 (10%)
NSAID	3 (7%)	2 (4%)	11 (18%)	6 (19%)

Except for PPI, no significant differences were detected between groups (p<0.017, Mann-Whitney non-parametric test with Bonferroni correction, compared to *H*. *pylori* resistance group). Abbreviations: PPI: Proton pump inhibitor, PI: Platelet inhibitor, NSAID: Non-steroidal anti-inflammatory drug.

### Upper GI symptoms

**Fig 1:**
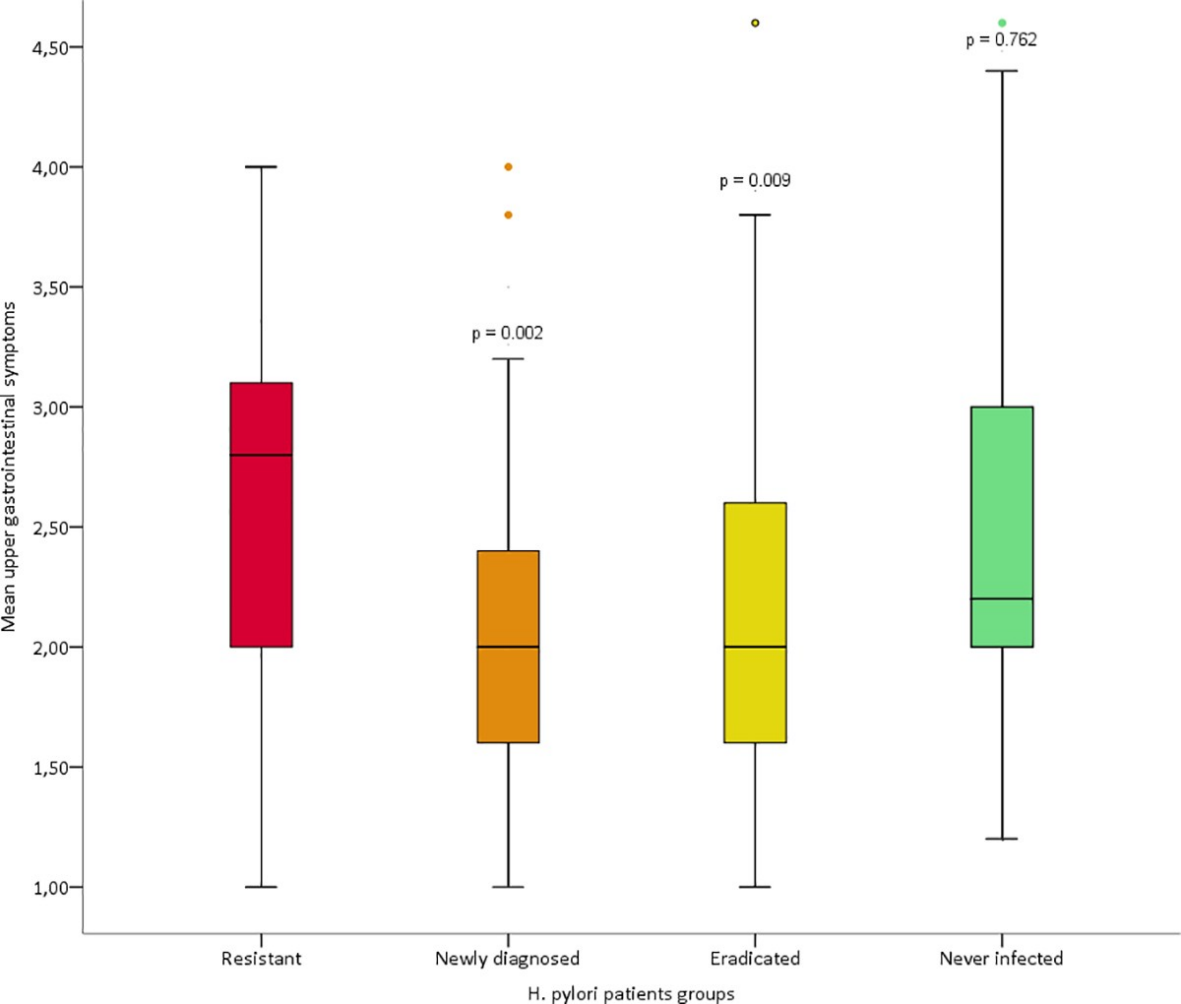
Baseline Gastrointestinal Symptoms Rating Scale (GSRS) scores in the five dimensions of upper gastrointestinal symptoms in the four different *H*. *pylori* groups. P-values represent comparison to the *H*. *pylori* resistant group.

The upper GI symptoms registered as total score of five dimensions (abdominal pain, indigestion, diarrhea, reflux and constipation [15,16]) in the five patient groups are shown in Table 2. The global test showed a p-value of 0.002 indicating a statistical significant difference in upper gastrointestinal symptom burden between the groups There was a significant difference in upper gastrointestinal symptom burden between groups. For the five dimensions of upper GI symptoms, the *H*. *pylori* resistant group showed a significant increased score compared to both the group of newly diagnostic *H*. *pylori* and *H*. *pylori* eradicated patients, but not to patients never infected with *H*. *pylori* (Table 2, Fig 1). In Table 2 each of the five symptom dimensions are also shown. No significant differences between the groups were observed regarding the prevalence of reflux syndrome and dyspepsia (Table 3).

**Table 2:** Baseline Gastrointestinal Symptoms Rating Scale (GSRS) of five dimensions of upper gastrointestinal symptoms in the four study groups.

*H*. *pylori* patient groups	Resistant	Newly diagnosed	Eradicated	Never infected
Abdominal pain	3.0 (2.6–3.4)	2.8 (2.4–3.1)	2.3 (2.0–2.5)**	3.3 (2.7–3.8)
Indigestion	3.1 (2.7–3.6)	2.3 (2.0–2.7)*	2.4 (2.1–2.6)	3.0 (2.8–3.8)
Diarrhoea	2.0 (1.5–2.4)	1.6 (1.3–2.0)	1.7 (1.5–2.0)	2.6 (1.9–3.3)**
Reflux	2.7 (2.2–3.2)	2.0 (1.7–2.3)	2.2 (1.9–2.4)	2.3 (1.8–2.8)
Constipation	2.2 (1.8–2.5)	1.7 (1.3–2.0)*	1.7 (1.4–2.0)*	2.1 (1.5–2.6)
Upper GI symptoms1	2.5 (2.3–2.8)	2.0 (1.7–2.2)*^,^***	2.1 (1.9–2.3)*	2.6 (2.2–2.9)

Values are presented as mean (95% CI).

1) Upper GI symptoms, calculated by mean sum of the 5 dimensions of the GSRS score as described in Materials and Methods.

*) compared to *H*. *pylori* resistant

**) compared to newly diagnosed *H*. *pylori*

***) compared to never *H*. *pylori* infected. p<0.017, Mann-Whitney U non-parametric test compared to *H*. *pylori* resistant group, with Bonferroni correction.

**Table 3:** Prevalence in the four study groups of functional bowel symptoms, reflux syndrome and dyspepsia as defined in Materials and Methods.

*H*. *pylori* patient groups	Resistant	Newly diagnosed	Eradicated	Never infected
FBS (n = 174)	28 (74%)	23 (48%)*	20 (34%)*	21 (72%)
Reflux syndrome (n = 182)	32 (78%)	29 (59%)	40 (66%)	23 (74%)
Dyspepsia (n = 184)	38 (90%)	46 (92%)	50 (82%)	30 (97%)

Numbers represent n (% of group). FBS: Functional bowel symptoms

*) p<0.017, Chi-Square test compared to *H pylori* resistant group, with Bonferroni correction.

### Functional bowel symptoms

**Fig 2:**
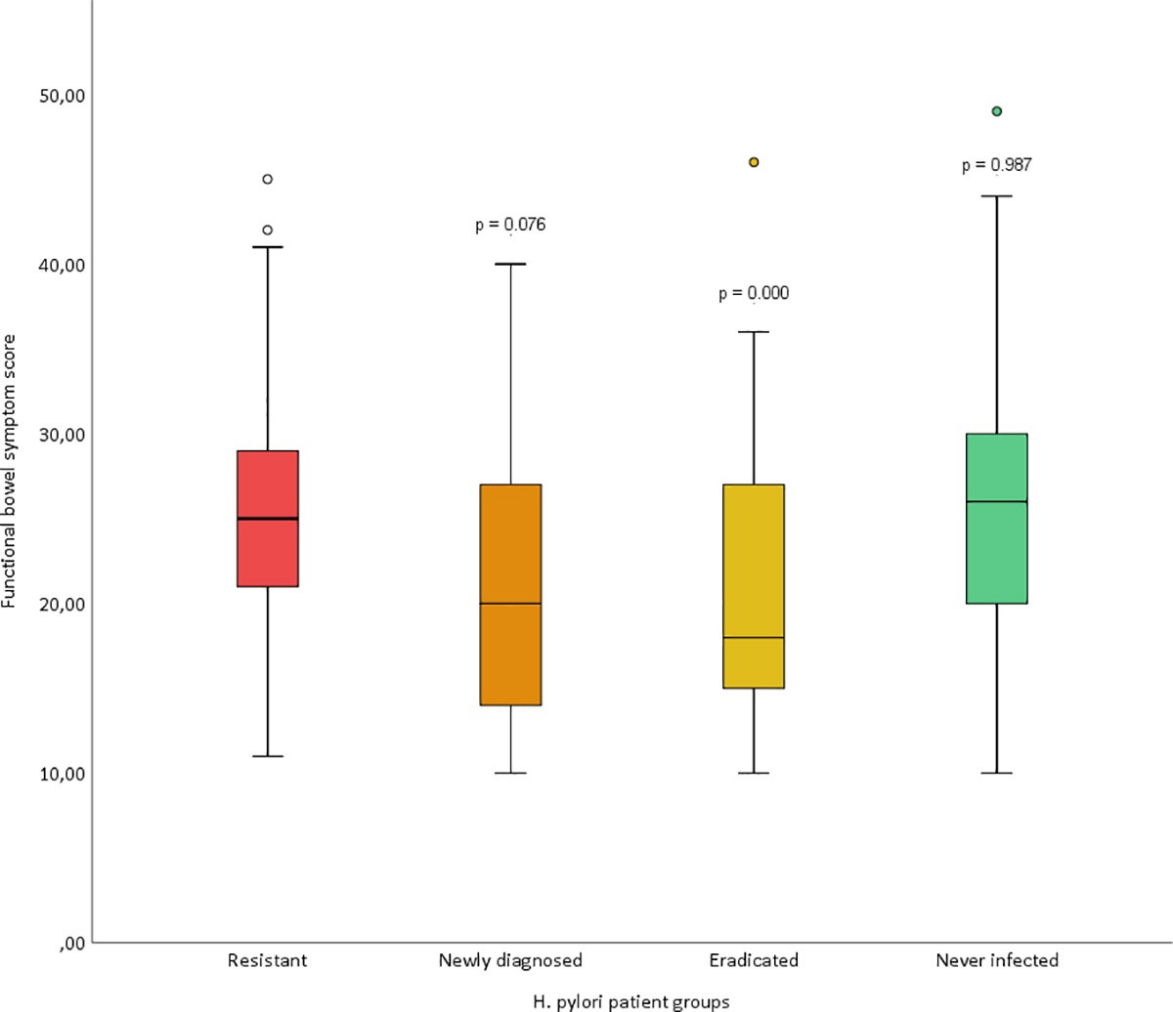
Functional bowel symptoms (FBS) based on baseline Gastrointestinal Symptoms Rating Scale (GSRS) of 5 dimensions as described in Materials and Methods in the four different *H*. *pylori* groups. P-values represent comparison to the *H*. *pylori* resistant group.

Functional bowel symptoms (FBS) based on GSRS dimensions that closest resemble irritable bowel syndrome according to Wicklund et al [19] are shown in Table 3 and Fig 2. FBS was more frequent observed in the *H*. *pylori* resistant group than in the newly diagnosed *H*. *pylori* and the *H*. *pylori* eradicated patients, but not when compared to patients never *H*. *pylori* infected.

### Endoscopic characteristics

The endoscopic findings are shown in Table 4. Gastritis was more prevalent in *H*. *pylori* positive patient groups compared to the *H*. *pylori* treated group and the *H*. *pylori* patient groups never infected group. Moreover, no significant differences were observed between the two infected *H*. *pylori* positive groups. No patients in any group had duodenal ulcer.

**Table 4:** Endoscopic characteristics of the four study groups.

*H*. *pylori* patient groups	Resistant	Newly diagnosed	Eradicated	Never infected
Normal	5 (12%)	6 (12%)	18 (30%)	18 (56%)
Gastritis	25 (60%)	31 (62%)	19 (31%)*	5 (16%)*
Esophagitis	3 (7%)	3 (6%)	3 (5%)	2 (6%)
Gastric Ulcer	1 (2%)	0	0	1 (3%)
Duodenitis	8 (19%)	9 (18%)	16 (26%)	4 (13%)
Hiatal hernia	0	1 (2%)	5 (8%)	2 (6%)

Values are presented as numbers (%).

*) p <0.017, Chi Square test with Bonferroni correction, compared to patients with active infection

### Histological characteristics

In Table 5, the Sydney histological scores are presented. The treatment resistant H- pylori patients had a lower score than the newly diagnosed *H*. *pylori* patient group. Moreover, the group of *H*. *pylori* eradicated patients and never *H*. *pylori* patient groups infected had significantly lower score that the two other groups.

**Table 5:** Sydney histological scores in gastric biopsies from the four study groups.

*H*. *pylori* patient groups	Resistant	Newly diagnosed	Eradicated	Never infected
All	2.3 (1.1) n = 42	3.2 (1.0)* n = 49	0.6 (0.9)*^,^** n = 61	0.4 (0.8)*^,^** n = 32
Before treatment	2.3 (2.0–2.7) n = 36	3.2 (2.9–3.3) n = 40	-	-
After treatment	1.1 (0.8–1.4) *** n = 36	1.4 (0.9–1.8)*** n = 40	-	-

The results are mean (SD) or mean (95% CI). Sydney score: Leucocyte infiltrate (total of neutrophils and monocytes) in gastric mucosa.

*), **) and ***): Significantly different (p<0.017 with Bonferroni correction) from *) *H*. *pylori* resistant, **) newly diagnosed and ***) before treatment.

In Table 6 the Ki-67 index and presence of atrophy in gastric biopsies are evaluated. For both parameters no significant differences were observed between the two *H*. *pylori* patient groups infected groups including the subgroups before and after eradication (total of 4 subgroups). Moreover, when comparisons were performed between each of these 4 groups to the group of previously *H*. *pylori* eradicated and the group never infected significant differences were observed and especially for Ki-67 as shown in Table 6. Significant difference between the *H*. *pylori* eradicated and never infected group was also shown in Ki-67 (medium-high). To be noted, there was no increased atrophy in the *H*. *pylori* resistant group compared to the newly diagnosed *H*, *pylori* patient groups diagnosed group nor the *H*. *pylori* patient groups eradicated group. Finally, atrophy was slightly reduced after *H*. *pylori* patient groups eradication in the two *H*. *pylori* patient groups infected groups but not at significant levels.

**Table 6:** Ki-67 and atrophy characteristics in the four study groups.

*H*. *pylori* patient groups	Resistant (before treatment)	Resistant (after treatment)	Newly diagnosed (before treatment)	Newly diagnosed (after treatment)	Eradicated	Never infected
Ki-67						
Low	3/42*^,^**	8/35*	6/45*	7/40*	18/58*	14/28
Medium-high	39/42*^,^**	27/35*	39/45*	33/40*	42/58*	14/28
Atrophy						
Normal	30/42	32/37	41/52	35/40	55/60	27/28
Low-high	12/42*^,^**	5/37	11/52	5/40	5/60	1/28

Numbers are ratios of scored biopsy specimens within each group.

*) and **): Significant different (p<0.017 with Bonferroni correction) from *) from: *) Never infected **) Eradicated. There were no significant differences between other groups.

### *H*. *pylori* patient groups, diagnostics

The *H*. *pylori* diagnostics (at least one positive test of *H*. *pylori*-urease test and a positive histological test of *H*. *pylori*) in the four different *H*. *pylori* patient groups groups are shown in Table 7. Histological *H*. *pylori* patient groups detection in biopsies was positive in a few biopsies with negative urease test.

**Table 7:** *H pylori* diagnostics in the four study groups.

*H*. *pylori* patient groups	All groups	Resistant	Newly diagnosed	Eradicated	Never infected
Positive urease test	82/184 (45%)	33/42 (79%)	49/49 (100%)	0/61 (0)	0/32 (0)
Positive histology	89/184 (48%)	40/42 (95%)	49/50 (100%)	0/61 (0)	0/32 (0)
At least one of two	92/184 (50%)	42/42 (100%)	50/50 (100%)	0/61 (0)	0/32 (0)

Values are presented as ratios (%).

### *H*. *pylori* patient groups, treatment

Thirty-six of the 42 patients in the *H*. *pylori* patient groups resistant group and 40 of the 50 patients in the newly *H*. *pylori* patient groups diagnosed group were treated according to the protocol. The Sydney score was significantly reduced in both treatment groups (Table 5)

## Discussion

We have made comparisons between a cohort of patients with treatment- resistance *H*. *pylori* infection and patients who underwent gastroscopy due to upper gastrointestinal complaints and who were further sub-grouped into “newly diagnosed *H*. *pylori* patient groups infection”, “previously *H*. *pylori* patient groups eradicated” and “never *H*. *pylori* patient groups infected”. The treatment-resistant cohort had a higher upper gastrointestinal symptom score, more functional bowel symptoms (FBS), and a reduced histological inflammation (Sydney) score compared to the newly diagnosed *H*. *pylori* patient groups group. Levofloxacin in combination with amoxicillin and omeprazole was highly effective in the eradication of *H*. *pylori* in the resistant patients and even more effective compared to newly *H*. *pylori* diagnosed patients treated with a conventional eradication regime (amoxicillin, clarithromycin and omeprazole). As far as we know this is the first broad characterization of patients with resistant *H*. *pylori* infection. Finally, the two non-infected groups had low Sydney scores, but only the never infected group–also defined as “true” functional dyspepsia–scored high both for upper gastrointestinal symptoms and FBS. This may imply two different clinical phenotypes.

The upper gastrointestinal symptom score was higher in the *H*. *pylori* resistant group compared to the group of newly diagnosed *H*. *pylori* infection, and even more increased compared to the previously (>5 years) eradicated *H*. *pylori* group. This score is based on the baseline Gastrointestinal Symptoms Rating Scale (GSRS) of five dimensions of upper gastrointestinal symptoms: abdominal pain, indigestion, reflux, diarrhoea and constipation, and the total score based on a study of patients referred to upper endoscopy for dyspepsia as validated in previous reports.[15,16] As seen for reflux in Table 2 and for reflux syndrome in Table 3 no significant differences were observed between the groups with and without active *H*. *pylori* infection. The role of *H*. *pylori* in reflux is also somewhat controversial, as both the presence and the absence of an association have been reported.[21–23] In a population-based study from our research group, *H*. *pylori* was protective against reflux symptoms in men.[24] The functional bowel symptom score was also increased in the *H*. *pylori* resistant group compared to the group of newly diagnosed *H*. *pylori* infection and even more increased compared to the previously *H*. *pylori* eradicated group. The association between *H*. *pylori* infection and IBS is not settled. *H*. *pylori* infection has been shown to be a risk factor for IBS in one study, but not in three other reports.[25–28] There are, however, no larger, population-based studies that have addressed this issue except from that reported by Breckan et al. in 2012 where *H*. *pylori* infection was not found to be associated with functional bowel symptoms.[20]

We used a combination of levofloxacin and amoxicillin in the second line triple therapy of *H*. *pylori*. This antibiotic combination has been reported to be successful in second-line *H*. *pylori* eradication with an efficacy of some 90%, as seen in our study.[11,13] An even higher score has been shown in sequential or concomitant use of levofloxacin, with a cumulative therapeutic efficacy of as high as 97.8% in second line therapy.[10,12] Despite the highly efficacy of levofloxacin and amoxicillin in second line triple therapy of *H*. *pylori* in our and other studies, it should be noticed that antibiotic resistance against *H*. *pylori* is an increasing worldwide health problem that now also includes levofloxacin resistance.[29] Therefore, the choice of antibiotics should be carefully selected to prevent antimicrobial resistance, and should be based on antibiotic resistance testing, including testing for levofloxacin according to the 2017 Maastricht Guidelines.[10]

In our study, gastric atrophy was more pronounced in the treatment resistant *H*. *pylori* group compared to the group never infected with *H*. *pylori*. This is in agreement with other reports including a meta-analysis by Adamu et al.[30] Moreover, *H*. *pylori* eradication reduced gastric atrophy as shown, yet only slightly. This may be explained by a short observation time after eradication (3 months). A meta-analysis by Rokka showed that *H*. *pylori* eradication indeed reduced gastric atrophy,[31] which implies that eradication of *H*. *pylori* in apparent treatment resistant cases should be given more priority in order to reduce which a proposed gastric precancerous condition.[32]

Of interest was the cohort of patients never infected with *H*. *pylori*—a group defined as “true” functional dyspepsia according the Maastricht V/Florence Consensus Report from 2017.[10] As expected, these patients presented both upper gastrointestinal symptoms and functional bowel symptoms (Tables 1 and 2, Figs 1 and 2) on a level with the *H*. *pylori* resistant patients. Conversely, the previously *H*. *pylori* eradicated patients had low Sydney and endoscopic gastric scores comparable to the never *H*. *pylori* infected, but this formerly infected group had low upper gastrointestinal symptom and functional bowel symptom scores. This may indicate that *H*. *pylori* infection, when being treatment resistant and/or infection being longstanding, may precipitate functional abdominal disorders including dyspepsia. One could propose that a successful eradication of *H*. *pylori* infection would reduce such gastrointestinal complaints. However, this awaits further studies.

The strength of this study is that we have performed a broad characterization of a *H*. *pylori* treatment resistant group and made comparisons to relevant patient groups with upper gastrointestinal symptoms. Moreover, we have tested for potential confounders when comparing groups of patients. The weakness of the study is that a follow-up study (>5 years after *H*. *pylori* eradication) has to be performed to characterize potential functional gastrointestinal symptoms.

In conclusion, patients with treatment-resistant *H*. *pylori* infection had more upper gastrointestinal symptoms and functional bowel symptoms, whereas the Sydney score was lower than in patients with newly diagnosed *H*. *pylori* infection. A triple combination of levofloxacin, amoxicillin and omeprazole showed a high efficacy in eradicating the infection.

## Supporting information

S1 File (TXT)
